# Actigraphy, the Alternative Way?

**DOI:** 10.3389/fpsyt.2014.00155

**Published:** 2014-12-03

**Authors:** Al W. De Weerd

**Affiliations:** ^1^Sleep-Wake Center SEIN, Zwolle-Groningen, Netherlands

**Keywords:** actigraphy, sleep disorders, polysomnography, MSLT, longtime recording

The gold standards for measurement of sleep and wake are polysomnography (PSG) and the multiple sleep latency test (MSLT) and will remain so. Both tests are labor intensive and expensive when seen from the viewpoint of the sleep laboratory and a heavy burden for the patient, in particular for children and their parents. Furthermore, the use of MSLT in children is hampered by the lack of reliable normal values.

Actigraphy is a simple technique. The actigraph looks like a small wristwatch and contains a device, which is sensitive to movements, a memory chip, and a battery. With a bin width that can be chosen dependent on the indication and the period of measurement, movements are detected and recorded by the memory chip. The idea is that during sleep only few movements will be detected in contrast to a period during wakefulness. The memory chip can be read through a simple device and plotted using a normal computer, notebook, or tablet. Parameters, such as time in bed (TIB), total sleep time (TST), sleep onset latency (SOL), awakenings and their duration, and naps during daytime, are calculated and showed automatically. As only little power is necessary, the actigraph can record continuously over long periods, which may extent to over 1 month in case the bin width is chosen conservatively, for example, every minute. Present day actigraphs record light intensity as well and some sophisticated models even record other parameters, such as heart frequency. The advantages when compared to the gold standards are obvious: easy to do for the laboratory, low cost (estimated at 5% of one PSG), no restrictions for the patient, and overview of a long period in one glance at the plot and quantification of important parameters of sleep and wake over the same period. The disadvantage lies in the presumption that little activity means sleep, and the lack of data on sleep stages, accompanying respiration disorders or abnormal movements as periodic limb movements, etc (1).

When compared on a day-to-day base, the data provided by actigraphy, differ with wide variations from the results of PSG on the parameters mentioned. In our study, in adults, the results are satisfactory and resembling, when the pooled mean results of at least five consecutive days of actigraphy (in our sleep center 1 week) is used for this comparison (Table 1). For this aspect in children, see Hyde et al. (2). Although only tested for long-term recording in patients with apnea syndrome, the device is approved and recommended by the AASM for an impression on sleep, but obviously not for the assessment of respiration. After many thousands of actigraphic measurement of at least 1 week, in our hands, the method provides similar results as mentioned in the AASM report. Our indications are far wider and actigraphy is used in our laboratory on a routine base in patients with insomnia, circadian rhythm disorders, apnea syndromes, restless legs syndrome, and hypersomnia. This holds for adults and children (Figure 1). In addition, assessment of the results of therapy for the sleep–wake disorder is easy using actigraphy, for example, in children with insomnia, limit setting disorder, and circadian rhythm disorders.

**Table 1:** **Correlation actigraphy/polysomnography**.

	TST^a^	SOL^a^	WASO^a^	SEI^b^
Range of diff./pat. (all nights)	−219 to +25	−21 to +209	−58 to +120	−34 to +54
Range of diff./pat. (mean of six nights)	−106 to +1	1 to 62	−19 to +48	−2 to +8
Mean diff./pat. (SD) (six nights)	−44 (28)	18 (7)	8 (3)	7 (7)

*N* = 22 patients with various sleep disorders

*^a^Minutes; ^b^%. All data calculated as PSG minus ACT*.

*ACT overestimates TST and underestimates the other parameters*.

*TST, total sleep time; SOL, sleep onset latency; WASO, wake after sleep onset; SEI, sleep efficiency*.

*Comparison of one night polysomnography (PSG) and mean of six nights actigraphy (ACT) resulting in 22 pairs for comparison*.

**Figure 1:**
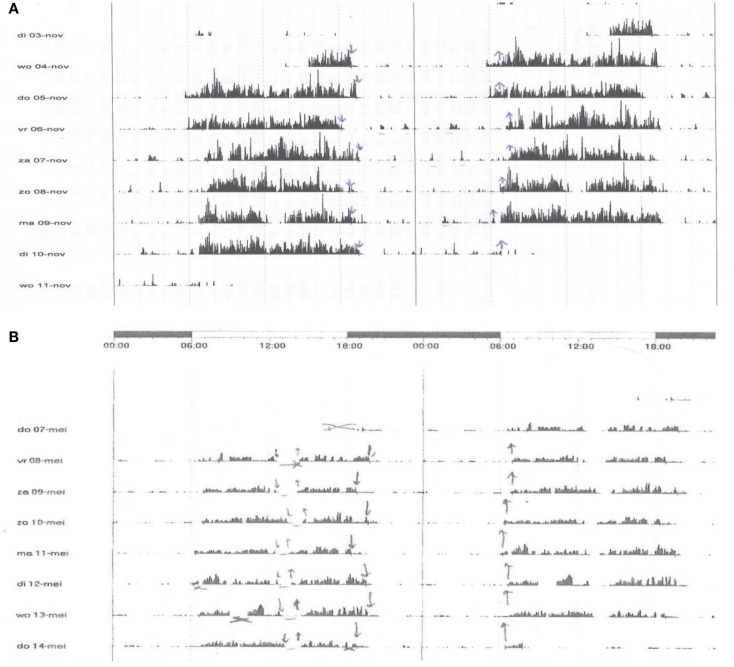

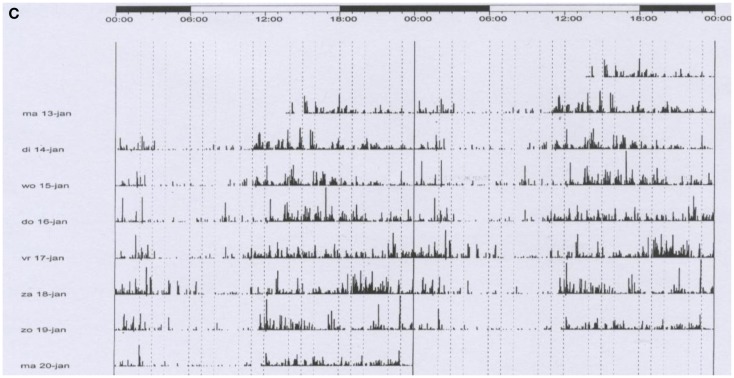
**The figure shows three registrations of actigraphy over a period of 7–8 days**. Each line represents 48 h. The plots are printed in a cascade mode: the right half of each line is printed as well as the left part of the next line in order to show clearly what happens around midnight. Arrows downward represent the moment of lights off; the upward arrows out of bed. **(A)** A boy, 3 years old, with insomnia and limit setting disorder. He sleeps from app. 7:30 p.m. to 6:30 a.m. Each night, he is very noisy when awake and terrorizes his parents. The actigraphy shows his regular bedtimes and the distinct periods of being active. Cognitive and behavioral therapy, in particular strict negation of the events during the night, was given to parents and child with good success. **(B)** A girl, 12 years old, complained about insomnia and tiredness during daytime. She felt sleepy in the afternoon. Actigraphy shows a prolonged sleep time of app. 12 h (7:00 p.m. to 7:00 a.m.) and regular naps after lunch. The differential diagnosis was long sleeper hypersomnia, bad sleep hygiene, or both. Narcolepsy was not expected due to lack of other phenomena, such as cataplexy and hallucinations. Polysomnography showed many spontaneous arousals and short awakenings. The main diagnosis was bad sleep hygiene. Cognitive and behavioral therapy resulted in a sleep period of app. 10 h when measured over the full 24 h. Sleep quality improved as did the tiredness. Naps were no longer necessary. This example shows as well that actigraphy has face value for the assessment of hypersomnia. **(C)** A boy 14 years of age. He ca not sleep before 2 o’clock in the morning and has severe problems to come out of bed before 11 a.m. Obviously, he fails at school, in particular in the first hours of morning classes. Actigraphy shows the real sleep/wake pattern. The diagnosis is Delayed Sleep Phase Syndrome. Cognitive and behavioral therapy, endorsed by light therapy, led to a satisfactory situation with timing of sleep between 11.30 p.m. and 8.15 a.m. This example shows the important role of actigraphy in the assessment of disturbances of the biological clock.

The device is in particular useful when used as an extension to a diagnostic PSG. In our lab, we perform PSG over two full days (thus including the wake time) together with actigraphy, followed by a period of at least 1 week of actigraphy only. In this way, we get the details on sleep and wake from PSG and a global overview over 9 days or more from actigraphy. As there is some overlap in time, the actigraphy gives background to the PSG data and vice versa. In a formal comparison of history, PSG over 24 h and actigraphy, the latter gives also reliable data on naps during daytime (Figure 1).

If PSG is not possible, actigraphy is a useful alternative. Patients with mental retardation, elderly patients who cannot come easily to the sleep laboratory, and (young) children are patient categories in whom actigraphy is an important way of recording sleep and wake. For children, our indications are insomnia, including limit setting disorder, hypersomnia, and parasomnia during the night. In teenagers and adolescents, the major indication is similar and includes the assessment of circadian rhythm disorders as well. The major restriction in these disorders is that actigraphy only gives an impression on the quality of sleep and wake, but never details of sleep (sleep stages, arousals, etc.), sleep disturbing factors as disorders of respiration or excessive movements (periodic limb movements), or the exact details of parasomnias. PSG and MSLT remain the gold standards, but actigraphy really helps when performed in the right way.

## Conflict of Interest Statement

The author declares that the research was conducted in the absence of any commercial or financial relationships that could be construed as a potential conflict of interest.
